# Discontinuation of tenofovir disoproxil fumarate from initial ART regimens because of renal adverse events: An analysis of data from four multi-country clinical trials

**DOI:** 10.1371/journal.pgph.0002648

**Published:** 2024-01-04

**Authors:** McNeil Ngongondo, Justin Ritz, Michael D. Hughes, Mitch Matoga, Mina C. Hosseinipour, on behalf of the AIDS Clinical Trials Group A5208, A5221, A5175 and A5274 Study Teams

**Affiliations:** 1 UNC Project Malawi, Lilongwe, Malawi; 2 Center for Biostatistics in AIDS Research, Harvard T. H. Chan School of Public Health, Harvard University, Boston, Massachusetts, United States of America; 3 The University of North Carolina School of Medicine, University of North Carolina at Chapel Hill, Chapel Hill, North Carolina, United States of America; University of Embu, KENYA

## Abstract

Tenofovir disoproxil fumarate (TDF), a potent and commonly used antiretroviral drug, is associated with renal tubular dysfunction and renal adverse events. We evaluated the frequency of, time to, and baseline risk factors for discontinuing TDF from initial antiretroviral therapy (ART) regimens because of renal adverse events from presumed tenofovir renal toxicity. We conducted an observational cohort study as a secondary analysis of data from four clinical trials conducted mainly in low- and middle-income countries. We included ART naïve participants living with HIV who started TDF-containing ART regimens in the trials. Participants had to have estimated creatinine clearance (eCrCl) equal to or greater than 60ml/min before starting ART. The primary outcome was the first instance of discontinuing TDF because of renal adverse events attributed to tenofovir renal toxicity during the first 48 weeks after starting ART. We evaluated the cumulative incidence of discontinuing TDF and associated risk factors using Fine and Gray competing risk regression models with a backward elimination variable selection strategy. There were 2802 ART-naïve participants who started TDF-containing ART from the four clinical trials were included in the analysis. Fifty-eight percent were female, the median age was 34 years, and 87% had CD4 cell counts less than 200 cells/μl. Sixty-four participants (2.4%, 95% CI 1.7%-2.8%) discontinued TDF due to renal adverse events. Among the 64 participants, the median time to discontinue TDF was 9.4 weeks (IQR: 3.4–20.7 weeks). From multivariable Fine and Gray regression models, risk factors for discontinuing TDF were older age, CD4 cell count <200 cells/μl, presence and severity of anemia, and eCrCl <90 ml/min. The risk of discontinuing TDF because of renal adverse events was low in participants initiating TDF-containing ART with advanced HIV and normal renal function, attesting to the tolerability of TDF in ART in low- and middle-income countries.

## Introduction

A durable and well-tolerated antiretroviral therapy (ART) regimen is an important goal when treating HIV infection [1, 2]. However, ART regimens can be modified for various reasons, including toxicity and intolerance, treatment failure, drug interactions, pregnancy, and patient or physician choice [3–5]. After an ART regimen has been modified, subsequent regimens are often associated with further modifications and poorer treatment outcomes, such as a higher risk of treatment failure and the acquisition of drug resistance [6, 7]. Furthermore, there are limited ART drug options in resource-limited settings [2]. Thus, modifying ART regimens poses challenges when treating HIV infection in resource-limited settings [2, 8].

Tenofovir disoproxil fumarate (TDF), a nucleoside reverse transcriptase inhibitor (NRTI) and a prodrug of tenofovir, is a widely used component of ART regimens [2, 9]. While it is effective and well tolerated, TDF is associated with renal toxicity that often requires its discontinuation from ART regimens [10–13]. Tenofovir alafenamide (TAF), a newer pro-drug formulation of tenofovir, has a more favorable renal safety profile [14, 15]. However, TDF continues to play a role in treating HIV. In the recent World Health Organization guidelines, TDF is still recommended for first-line ART regimens [2]. It is also widely used in preexposure and postexposure prophylaxis [2]. It is important to understand how TDF is discontinued because HIV care providers frequently interrupt ART and discontinue TDF when they suspect any renal disease since it is usually impossible to rule out some contribution of TDF towards the renal disease [2, 16].

In this study, we evaluated the discontinuation of TDF from initial ART regimens attributed to presumed tenofovir-associated renal toxicity and renal adverse events in four multi-country clinical trials from the AIDS Clinical Trials Group (ACTG). These clinical trials were done primarily in low- and middle-income countries. We evaluated the frequency of, time to, and baseline risk factors for discontinuing TDF.

## Methods

### Study design and population

We analyzed data from four randomized prospective clinical trials. The clinical trials were conducted between 2005 and 2016 (Table 1). All four trials enrolled ART-naïve participants who started initial ART regimens in the clinical trials. Participants generally started study-provided ART. In some cases, and at the study clinicians’ discretion, participants started locally available ART. The clinical trials are:

1. Prospective Evaluation of Antiretrovirals in Resource-Limited Settings (PEARLS) trial (AIDS Clinical Trials Group (ACTG) A5175, NCT00084136) evaluated the efficacy of ART regimens consisting of two nucleoside reverse transcriptase inhibitors (NRTIs) + a protease inhibitor (PI) and two NRTIs + a non-nucleoside reverse transcriptase inhibitor (NNRTI).
2. Optimal Combination Therapy after Nevirapine Exposure (OCTANE) trial (ACTG A5208, NCT00089505) comprised two trials that were conducted concurrently: Trial 1 evaluated the superiority of PI-based ART over NNRTI-based ART in women with prior single-dose nevirapine (NVP) prophylaxis, while Trial 2 compared PI- and NRTI-based ART in women with no previous NVP exposure.
3. A Strategy Study of Immediate Versus Deferred Initiation of Antiretroviral Therapy for AIDS Disease-Free Survival in HIV-Infected Persons Treated for Tuberculosis with CD4 Less Than 250 Cells/mm^3^ (STRIDE) trial (ACTG 5221, NCT00108862) evaluated whether immediate versus deferred initiation of ART reduces mortality and AIDS-defining events in participants on tuberculosis (TB) treatment. Participants were randomized to receive either ART within 14 days after starting TB treatment or to have ART deferred until 8–12 weeks after starting TB treatment.
4. Reducing Early Mortality & Morbidity by Empiric Tuberculosis (TB) Treatment (REMEMBER) trial (ACTG A5274, NCT01380080) assessed whether empirical TB treatment reduced early mortality in participants with advanced HIV disease living in high-burden settings compared with isoniazid preventive therapy (IPT). Participants were randomized to receive either IPT plus ART or TB treatment plus ART for 24 weeks. After 24 weeks, ART was continued all participants.

**Table 1:** Characteristics of studies included in the analysis.

Study	Period of study	Initial TDF-containing ART regimen	Number that initiated TDF-containing ART	Renal indication for discontinuing TDF*
**PEARLS**	2005–2010	EFV/FTC/TDF	525	eCrCl <50 ml/min sCr >1.5-fold above baseline Hypophosphatemia
**OCTANE**	2006–2011	NVP+FTC/TDF	368	eCrCl <50 ml/min
		LPV/r+FTC/TDF	370	
**STRIDE**	2006–2010	EFV/FTC/TDF	753	eCrCl <50 ml/min
**REMEMBER**	2011–2016	EFV/FTC/TDF	786	eCrCl <50 ml/min

*All studies allowed interruption of TDF for clinical decisions suspecting tenofovir toxicity

Abbreviations: eCrCl, estimated creatinine clearance; SCr, serum creatinine; PEARLS, Prospective Evaluation of Antiretrovirals in Resource-Limited Settings; OCTANE, Optimal Combination Therapy after Nevirapine Exposure; STRIDE, A Strategy Study of Immediate Versus Deferred Initiation of Antiretroviral Therapy for AIDS Disease-Free Survival in HIV-Infected Persons Treated for Tuberculosis with CD4 Less Than 250 Cells/mm^3^; REMEMBER, Reducing Early Mortality & Morbidity by Empiric Tuberculosis (TB) Treatment (REMEMBER) trial

We restricted this analysis to a subset of participants in the four clinical trials who started TDF-containing ART and restricted the period for the analysis to the first 48 weeks of ART. In all four clinical trials, participants starting TDF-containing ART were required to have an estimated creatinine clearance (eCrCl) equal to or greater than 60ml/min. The studies used the Cockcroft-Gault equation to calculate the eCrCl [17]. The clinical trials were conducted in an era where ART was started based on CD4 cell count thresholds and before the test and treat approach.

Table 1 shows a summary of the clinical trials.

This analysis includes participants recruited from sites in 14 countries in Africa, Asia, North America, and South America. Sites obtained ethical approval from their ethics committees. All participants provided written informed consent.

### Data collection

In all studies, participants had a study entry visit at which demographic information, clinical assessments including weight and height, full blood counts, serum chemistry including serum creatinine, CD4+ cell count, and plasma HIV RNA were collected. After study entry, study visits were scheduled at least every 12 weeks, at which clinical assessments, serum chemistries, including serum creatinine and urinalysis were done.

Interruptions to ART regimens were reported on a standard case report form (CRF) used in all four clinical trials. Study clinicians evaluated participants for adverse events at scheduled study visits or at interim clinic visits if participants presented with an illness outside the scheduled study visit. Study clinicians assessed any suspected relatedness of adverse events, including renal adverse events, to individual antiretroviral drugs and recorded any actions taken on the antiretroviral drugs to the CRF.

### Measures

#### Outcome

The primary study outcome for this analysis was the discontinuation of TDF from initial TDF-containing ART regimens due to renal adverse events presumed to have been caused by tenofovir renal toxicity within 48 weeks of starting ART. This determination was made by study clinicians using their clinical judgment and guided by the toxicity management sections of the study protocols and in consultation with Clinical Management Committees for each trial. The study protocols required discontinuation of TDF following confirmed eCrCl <50mL/min, grade 3 or 4 increases in serum creatinine, or grade 4 hypophosphatemia. The outcome definition encompasses actions taken to modify the ART regimen, including permanently discontinuing and replacing the ART regimen with a different ART regimen that did not contain TDF.

#### Covariates

We assessed the following variables at study entry as potential risk factors for discontinuing TDF due to renal adverse events: age, sex, CD4 cell count, HIV viral load, body mass index (BMI), presence and severity of anemia, eCrCl and source clinical trial for the participants.

We categorized age according to the distribution of ages in the study population into <30 years, 30–44 years, and ≥45 years. We categorized CD4 cell count according to the WHO immunological classification for established HIV infection into <200 cells/μl (severe immunodeficiency) and ≥200 cells/μl (moderate, mild, and not significant immunodeficiency) [18]. We categorized BMI according to the WHO classification of BMI into <18.5kg/m^2^ (underweight), 18.5–24.9 kg/m^2^ (normal weight), and >24.9kg/m^2^ (overweight) [19]. We categorized hemoglobin levels according to the WHO hemoglobin concentrations for the diagnosis of anemia and assessment of severity into >12.0 g/dl (normal hemoglobin in women) and >13.0 g/dl (normal hemoglobin in men), 11–11.9 g/dl (mild anemia in women), 11–12.9 g/dl (mild anemia in men), 8–10.9 g/dl (moderate anemia in both sexes) and <8 g/dl (moderate anemia in both sexes) [20]. We categorized eCrCl into <90ml/min (low) and ≥90ml/min (normal) as a reflection of how creatinine clearance is used in clinical practice [21].

### Statistical analysis

We summarized the characteristics of participants using frequency and percentages.

We calculated the incidence proportion of discontinuing TDF as the proportion of participants who discontinued TDF-containing ART due to renal adverse events within 48 weeks of starting ART.

In a time-to-event analysis, the origin of follow-up was the date of starting a TDF-containing ART regimen. Participants who did not discontinue their TDF-containing ART were censored at 48 weeks (i.e. administrative censoring). We treated instances of discontinuation of TDF-containing ART for reasons other than renal adverse events, i.e., other adverse events, clinical reasons, and death, as competing events.

We used Fine and Gray competing risk regression models to select a model with variables predicting discontinuation of TDF due to renal adverse events in the presence of competing events [22, 23]. First, we assessed univariate associations between the time to discontinuation of TDF and each potential risk factor. All variables included in the multivariable regression analysis had a p-value <0.25 [24]. In the multivariable regression analysis, we used a backward elimination variable selection method to identify the most important risk factors for the discontinuing TDF [23, 25]. We set the p-to-remove variables in the models at p-value <0.15 [24]. We set a higher significance level for variable selection to avoid missing important risk factors relevant to the outcome and to capture clinically significant variables that may have less statistical significance [24]. We checked the proportionality assumption required for the validity of the models by including an interaction term with time in a model for each covariate separately and assessing the statistical significance of the interaction term at α = 0.05.

We used a Fine and Gray regression model to derive an unadjusted cumulative incidence function which was used to create a cumulative incidence curve showing the risk of discontinuing TDF during the 48 weeks of follow-up.

We used a complete case analysis because there was minimal missing data. Only 30/2802 (1%) of participants had missing covariate data (Table 1 below).

We performed all analyses using SAS software version 9.4 (SAS Institute, Cary, NC, USA).

## Results

A total of 2802 (female = 58%) participants were included in this analysis. The median age was 34 years (IQR: 29–40 years). Eighty-seven percent had severe immunosuppression with CD4 cell counts <200 cells/μl. Twenty-three percent were underweight, having a BMI <18.5 kg/m^2^. Seventy percent had anemia: mild (26%), moderate (38%), and severe (5%) (Table 2).

**Table 2:** Baseline characteristics of participants who started TDF-containing ART regimens in the PEARLS, OCTANE, STRIDE, AND REMEMBER clinical trials.

Characteristics at study entry	Discontinuation of TDF due to renal adverse event		
	Yes, n (%)	No, n (%)	Total, n (%)
	N = 64	N = 2738	N = 2802
**Sex**			
Male	27 (2.3)	1145 (97.7)	1172 (41.8)
Female	37 (2.3)	1593 (97.7)	1630 (58.2)
**Age (years)**			
<30	15 (2.0) 1.98	742 (98.0)	757 (27.0)
30–44	32 (1.9)	1634 (98.1)	1666 (59.5)
≥ 45	17 (4.5)	362 (95.5)	379 (13.5)
**CD4 cell count (cells/μl)**			
<200	62 (2.5)	2383 (97.5)	2445 (87.3)
≥200	2(0.6)	355 (99.4)	357(12.7)
**Body mass index (kg/m^2^)**			
<18.5	25 (3.8)	631 (96.2)	656 (23.4)
18.5- <25	32 (1.9)	1615 (98.1)	1647 (58.8)
≥25	7 (1.4)	492 (98.6)	499 (17.8)
**Anemia** *			
None	7 (0.8)	865 (99.2)	872 (31.2)
Mild	10 (1.4)	719 (98.6)	729 (26.1)
Moderate	39 (3.7)	1014 (96.3)	1053 (37.6)
Severe	8 (5.5)	137 (94.5)	145 (5.2)
**eCrCl (ml/min)** **			
≤90	37 (4.0)	884 (96.0)	921 (33.1)
>90	27 (1.5)	1835 (98.6)	1862 (66.9)
**Study**			
PEARLS	5 (1.0)	520 (99.1)	525 (18.7)
OCTANE	12 (1.6)	726 (98.4)	738 (26.3)
STRIDE	21 (2.8)	732 (97.2)	753 (26.9)
REMEMBER	26 (3.3)	760 (96.7)	786 (28.1)
**HIV RNA copies/ml** ***			
≤100,000	15 (1.6)	949 (98.4)	964 (34.5)
>100,000	49 (2.7)	1780 (97.3)	1829 (65.5)

*Missing n = 3

**Missing n = 18

***Missing n = 9

Abbreviations: ART, antiretroviral therapy; BMI, body mass index; eCrCl, estimated creatinine clearance; PEARLS, Prospective Evaluation of Antiretrovirals in Resource-Limited Settings; OCTANE, Optimal Combination Therapy after Nevirapine Exposure; STRIDE, A Strategy Study of Immediate Versus Deferred Initiation of Antiretroviral Therapy for AIDS Disease-Free Survival in HIV-Infected Persons Treated for Tuberculosis with CD4 Less Than 250 Cells/mm3; REMEMBER, Reducing Early Mortality & Morbidity by Empiric Tuberculosis Treatment.

**Fig 1:**
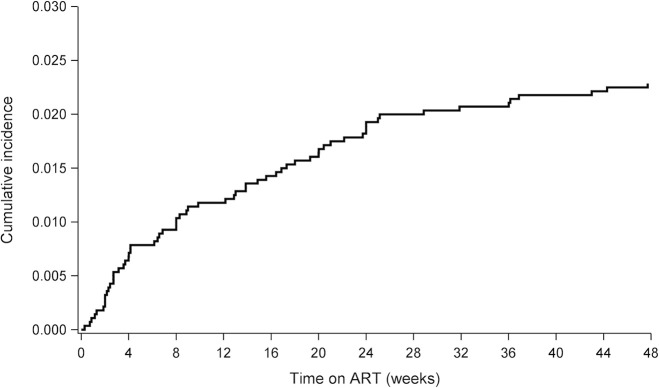
Cumulative incidence curve of discontinuing TDF within 48 weeks after starting ART.

Sixty-four participants (2.3%, 95% CI 1.7%-2.8%) discontinued TDF due to renal adverse events within 48 weeks of starting ART in the four clinical trials. Fig 1 presents a cumulative incidence curve showing the cumulative probability of discontinuing TDF due to renal adverse events and the distribution of the events over the 48 weeks of follow-up. Most of the discontinuations occurred early during follow-up: 52% occurred by week 12 and 84% by week 24 of starting TDF-containing ART. Among the 64 participants, the median time to discontinue TDF was 9.4 weeks (IQR: 3.4–20.7).

The renal adverse events that led to discontinuation of TDF were decreased eCrCl or increased serum creatinine (61 participants) and hypophosphatemia (3 participants).

Through the backward elimination variable selection procedure, the final model included four variables as risk factors for discontinuing TDF due to renal adverse events ‒ higher age, presence, and severity of anemia, lower baseline eCrCl, and lower CD4 cell count. The presence and severity of anemia and lower eCrCl were statistically significant at α = 0.05 significance level and were the strongest risk factors for discontinuing TDF. Moderate (HR = 3.8, 95% CI: 1.7–8.7) and severe anemia (HR = 5.6, 95% CI: 2.0–15.7) and eCrCl <90 ml/min (HR = 2.3, 95% CI: 1.3–3.9) were associated with a higher risk of discontinuing TDF (Table 3).

**Table 3:** Unadjusted and adjusted hazard ratios and 95% CIs for the association between baseline characteristics and discontinuation of TDF from TDF-containing ART regimens in participants in the PEARLS, OCTANE, STRIDE, and REMEMBER clinical trials.

Characteristic	Unadjusted HR (95% CI)	P-value	Adjusted HR (95% CI)	P-value
		
**Age (years)**				
<30 (ref)	1	0.011	1	0.10
30–44	1.0 (0.5, 1.8)		0.9 (0.5–1.6)	
≥ 45	2.3 (1.1, 4.6)		1.7 (0.9–3.6)	
**Sex**				
Male (ref)	1	0.96		
Female	1.0 (0.6, 1.6)			
**CD4 cell count (cells/μl)**				
≥200 (ref)	1	0.034	1	0.09
<200	4.6 (1.1, 18.7)		3.5 (0.8–14.8)	
**Body mass index (kg/m^2^)**				
<18.5 (ref)	1	0.011		
18.5- <25	0.5 (0.3, 0.9)			
≥25	0.4 (0.2, 0.8)			
**Anemia**				
None (ref)	1	<0.001	1	<0.001
Mild	1.7 (0.7, 4.5)		1.4 (0.5–3.7)	
Moderate	4.7 (2.1, 10.5)		3.8 (1.7–8.7)	
Severe	7.1 (2.6, 19.7)		5.6 (2.0–15.7)	
**eCrCl (ml/min)**				
≥90 (ref)	1	<0.001	1	0.002
<90	2.8 (1.7, 4.6)		2.3 (1.3–3.9)	
**ACTG study**				
PEARLS (ref)	1	0.026		
OCTANE	1.7 (0.6, 4.9)			
STRIDE	3.0 (1.1, 7.9)			
REMEMBER	3.5 (1.4, 9.1)			
**HIV RNA (copies/ml)**				
≤100,000 (ref)	1	0.063		
>100,000	1.7 (1.0, 3.1)			

Abbreviations: HR, Hazard ratio; CI, confidence interval; ref, reference level; eCrCl, estimated creatinine clearance; PEARLS, Prospective Evaluation of Antiretrovirals in Resource-Limited Settings; OCTANE, Optimal Combination Therapy after Nevirapine Exposure; STRIDE, A Strategy Study of Immediate Versus Deferred Initiation of Antiretroviral Therapy for AIDS Disease-Free Survival in HIV-Infected Persons Treated for Tuberculosis with CD4 Less Than 250 Cells/mm3; REMEMBER, Reducing Early Mortality & Morbidity by Empiric Tuberculosis Treatment

## Discussion

In this analysis, we found a low risk of discontinuing TDF from initial TDF-containing ART regimens following renal adverse events during the first 48 weeks of ART. Most of the discontinuations occurred early, in the first 12 weeks of treatment. Older age, CD4 cell counts <200 cells/ml, the presence and severity of anemia, and eCrCl <90 ml/min were associated with a higher risk of discontinuing TDF from the ART regimens.

The proportion of people who discontinued TDF in this study, at 2.3%, is consistent with prior evidence showing a low risk of renal adverse events attributed to TDF and, subsequently, a low risk of discontinuation of TDF due to renal adverse events observed during ART [12, 13, 26]. Many studies and millions of accumulated years of using TDF in ART support the safety and durability of TDF-containing ART [11, 12]. On some occasions, discontinuing TDF reflects practitioners’ anticipation of and caution towards renal adverse events rather than actual tenofovir-associated renal tubular toxicity for which tenofovir would be the offender [13]. Often, there are other causes of renal dysfunction other than tenofovir, such as HIV-associated nephropathy, opportunistic infections, hypertension, diabetes mellitus, and other medications [27, 28].

Our study population was particularly high-risk for renal dysfunction because the four contributing clinical trials enrolled a high proportion of participants with severe immunosuppression (87%) [16, 29]. Thus, the study showed a trend toward higher risk among those with CD4 cell count <200 cells/μl. Clinical trials that enroll healthier, more stable participants report fewer or no discontinuations of TDF due to renal adverse events. In an analysis of 21 clinical trials in which ART-naïve individuals started TDF-containing ART, the proportion discontinuing TDF was only 0.43% [13]. In other clinical trials, there were no discontinuations of TDF due to renal adverse events at all [30–32]. The higher discontinuation rate identified in this analysis is likely due to the inclusion of a high-risk population. Nevertheless, the study confirms that the risk of discontinuing TDF is low [13, 16].

Renal function tests are not readily available in many resource-limited settings. In these settings, HIV is often treated without laboratory monitoring [33]. The DART study showed that routine renal function monitoring during ART was unnecessary because it did not benefit clinical outcomes [34]. In the DART trial, routine renal function monitoring did not change the time to modification of ART due to adverse events, including in 75% of participants who were on TDF-containing ART [34]. Instead, the DART study findings, like the WHO recommendations, support targeted renal function testing and monitoring in people at risk of or suspected of renal dysfunction [2, 34]. Individuals at high risk for renal dysfunction should avoid TDF-containing ART by using alternative ART [2, 33, 34].

TAF, an alternate prodrug of tenofovir, has a more favorable renal safety profile than TDF. In several studies comparing TAF- and TDF-containing ART, there were no discontinuations of TAF for renal tubular toxicity in contrast to TDF [14, 35]. TAF results in lower plasma concentrations of tenofovir compared to TDF, thus it can be given in smaller doses and reduces tenofovir exposure to the kidneys [15]. Therefore, TAF has the same clinical efficacy as TDF, with a much more favorable renal safety profile [15]. Crucially, TAF requires less laboratory monitoring of renal function [15, 36]. Currently, TDF continues to play a significant role in treating HIV as it is a recommended NRTI in first-line ART regimens in WHO HIV treatment guidelines [2].

By drawing from several clinical trials, the study population in this analysis is diverse. It includes a large proportion of women and substantial geographic representation. Thus, a strength of this study is that the results can be generalized to diverse populations typical in clinical practice [11, 37]. Another strength of the study is that the definition of renal adverse events, reporting of the adverse events, and management of the ART regimens were similar in the four clinical trials, as they followed similar renal toxicity management protocols (all clinical trials are from the same study consortium, the ACTG). This uniform assessment of adverse events across the clinical trials minimized any misclassification of renal adverse events and misattribution of the reason for discontinuing TDF. An additional strength of this analysis is that participants in the clinical trials were new ART users. This avoids the bias of underestimating adverse events in prevalent users of a medication [38].

A limitation of this analysis is that we did not have good data on tubular markers of renal tubular toxicity, such as metabolic acidosis with normal plasma ion gap, hyperphosphaturia, hypokalemia, hypouricemia, urinary tubular protein waste, glycosuria with normal blood glucose, and aminoaciduria [39]. Tubular toxicity is specific to TDF, and its markers would indicate that the presumed renal adverse events that led to discontinuation of TDF were more accurately attributed to TDF rather than other pathologies. These other pathologies include HIV-associated nephropathy, diabetes mellitus, hypertension and other drugs. Having tubular markers of renal tubular toxicity would reduce misclassification of the outcome by increasing the specificity of classifying discontinuation as TDF-related.

Another limitation is that we did not evaluate PIs as a potential risk factor for discontinuing TDF. PIs have been associated with decreased renal function during ART, especially when the PIs are combined with TDF [40, 41]. Among the four clinical trials, the OCTANE study is the only one that had a TDF/PI combination as an initial ART regimen. In that study, there was no difference in discontinuation of ART when TDF was combined with lopinavir/ritonavir versus with an NNRTI [42]. Because the three other studies in the analysis included only NNRTI combinations with TDF, we did not conduct further analysis stratified by the type of ART.

The overall durability of ART depends not only on renal adverse events but also on other reasons for modifying ART. Other common reasons for modifying ART include other drug toxicities, treatment failure, availability, poor adherence to ART, concomitant clinical conditions, and patient or physician choice to simplify treatment [1, 43–45]. We did not look at other reasons for modifying ART, focusing only on renal adverse events.

Predicting which patients may experience renal toxicity and discontinue TDF in the future is challenging [46]. While the risk factors we identified may not be predictive of future TDF discontinuation, they could be used to categorize ART naïve participants as ‘high’ or ‘low’ risk. This could be used to determine whether an individual is more closely monitored during the initial months of a TDF-containing ART regimen.

### Conclusions

In conclusion, we have shown that the proportion of patients who had TDF discontinued from their ART regimen due to presumed renal adverse events was low during the first year of treatment. We found that the risk factors for discontinuing TDF were older age, presence and severity of anemia, low baseline eCrCl, and CD4 cell count <200 cells/μl. Generally, each of these variables are routinely available to ART providers as part of the standard of care when starting ART. Additionally, these variables are easily understood by patients. This can help with counseling patients about TDF, and with monitoring and managing ART during treatment.
